# Impact of pharmacist-led aminoglycoside stewardship: a 10-year observational study

**DOI:** 10.1186/s40780-024-00399-x

**Published:** 2024-11-28

**Authors:** Yasutaka Shinoda, Kengo Ohashi, Tomoko Matsuoka, Kaori Arai, Nao Hotta, Eiseki Usami

**Affiliations:** Department of Pharmacy, Ogaki Municipal Hospital, Gifu, Japan

**Keywords:** Aminoglycosides, Endocarditis, Therapeutic drug monitoring, Antimicrobial stewardship, Pharmacist

## Abstract

**Background:**

Aminoglycosides are crucial for treating multidrug-resistant gram-negative infections and endocarditis. However, aminoglycosides are associated with significant risks of nephrotoxicity, necessitating careful dose selection and therapeutic drug monitoring. Therapeutic drug monitoring is essential for minimizing risk; however, few institutions routinely perform it. This study aimed to assess the impact of a pharmacist-driven therapeutic drug monitoring intervention on aminoglycoside usage trends and clinical outcomes.

**Methods:**

This retrospective cohort study included 263 patients treated with aminoglycosides between 2014 and 2023. A pharmacist-led therapeutic drug monitoring intervention began in 2017, focusing on monitoring renal function, documenting patient weight, and closely managing aminoglycoside concentrations. Trends in aminoglycoside use and renal outcomes were analyzed.

**Results:**

Over the study period, appropriate use of aminoglycosides at the time of initial prescription increased from 49 to 82% (*P* < 0.01). Pharmacist dosing design at initial prescription increased significantly from 21% pre-intervention to 60% post-intervention (*P* < 0.01). The proportion of pharmacist intervention in initial dosing design increased over time. The proportion of patients with measured aminoglycoside blood concentrations significantly increased from 53% pre-intervention to 72% post-intervention (*P* < 0.01). The proportion of patients who were able to manage target blood concentrations from the initial aminoglycoside dose without dose adjustments increased from 31% pre-intervention to 42% post-intervention, although the results were not significantly different (*P* = 0.07). The incidence rate of renal impairment remained similar (11% vs. 12%; *P* = 0.85), although the annual average number of cases decreased from 4.3 before the intervention to 2.5 after. Similarly, there were no significant differences in clinical efficacy before and after the intervention (65% vs. 71%; *P* = 0.35). Furthermore, aminoglycoside stewardship led to a 56% cost saving.

**Conclusions:**

Pharmacist-led aminoglycoside stewardship significantly improved the appropriate use of aminoglycosides and decreased the associated costs. Thus, pharmacist involvement is essential for the proper use of aminoglycosides. However, many patients required aminoglycoside dose reductions despite the pharmacist’s guideline-based dosing design. Therefore, further accumulation of information on the management of aminoglycoside blood concentration may be necessary for the revision of these guidelines.

**Supplementary Information:**

The online version contains supplementary material available at 10.1186/s40780-024-00399-x.

## Background

Aminoglycosides (AGs) are essential antibiotics used to treat multidrug-resistant gram-negative bacterial infections and other infections, such as endocarditis [1–4]. However, AGs carry a high risk of nephrotoxicity, necessitating careful dose selection and therapeutic drug monitoring (TDM) to balance efficacy and safety [5, 6]. Nephrotoxicity reportedly occurs in 6–58% of patients treated with AGs, emphasizing the need for routine TDM [7–11].

TDM of antimicrobial agents is an important field for pharmacists. Joint guidelines from the Infectious Disease Society of America and the American Society for Healthcare Epidemiology recommend incorporating stewardship in the form of TDM as routine practice in hospital pharmacy departments [12]. Although TDM is the core responsibility of pharmacists, only approximately 10% of institutions consistently implement TDM for AGs [13]. Some reports indicate that only 4% of hospitals adhered to general TDM guidelines for the use of gentamicin (GM) [14]. Therefore, evaluating the effectiveness of pharmacist-led TDM for optimizing AG use is crucial.

This study aimed to assess the impact of a pharmacist-driven TDM intervention implemented at our institution in 2017 on AG usage trends and clinical outcomes.

## Methods

### Study population

Patients treated intravenously with tobramycin, amikacin (AMK), GM, or arbekacin were included in this study. Patients undergoing renal replacement therapy, those with < 2 days of treatment, and those aged < 18 years were excluded. The study period ranged from January 1, 2014, to December 31, 2023, with the pharmacist-led TDM intervention starting on January 1, 2017. Medical records were retrospectively examined to assess the effects of this intervention.

### Ethics approval

This study was approved by the Ethics Committee of our hospital (approval number: 202401). Further, this study was conducted with comprehensive opt-out consent obtained from all participants.

### Pharmacist-led intervention

The following pharmacist-led TDM intervention (hereinafter referred to as “the intervention”) was initiated on January 1, 2017. First, pharmacists identified patients with new prescriptions for AGs during the weekday day shift. Second, all newly prescribed patients were charted for the need for AGs and recommendations for appropriate dosage and blood concentration measurements. Third, physicians were contacted by phone if there was no justification for AG use or if dosage deviations occurred. Fourth, follow-up with the patients was performed every weekday during the day shift to determine if AGs were required and if there were any TDM issues; consultation was performed with the physician by phone, chart notes, and the ward pharmacist, as required.

In this study, before the intervention, the pharmacist provided feedback on the simulation results only when blood concentrations were measured at the physician’s discretion. Moreover, a physician’s order to measure blood concentrations was conducted to measure peak and trough concentrations.

### Evaluation of outcomes

This study retrospectively compared AG use before and after the intervention. The appropriateness of AG dosing was evaluated based on weight documentation, renal function monitoring, and infection site or pathogen considerations. In this study, adjusted body weight was used to assess AG dose adequacy in patients with ≥ 20% of their ideal body weight (IBW), as follows: IBW = A + 0.91 x [height – 152.4], where A is 50 for men and 49.5 for women; and adjusted body weight (kg) = IBW + [0.4 x (actual body weight - IBW)]. In addition, based on adopted formulation standards and clinical practices, appropriate dose tolerances were less than ± 100 mg for AMK and less than ± 60 mg for GM, tobramycin, and arbekacin (Additional File 1, Table S1). When the minimum inhibitory concentration of the causative organism was unknown, the dose was considered appropriate if within the range based on the patient’s renal function (Additional File 1, Table S1). AG blood concentrations were considered adequate when both peak and trough concentrations were within the recommended range. When only one of these concentrations was measured due to an error during collection, it was excluded from adequacy determination in this study.

AG usage was categorized into three levels: “Recommended,” “Optional,” and “Not recommended,” based on existing guidelines [1, 2, 15–18], including the Sanford Guide for the Treatment of Infectious Diseases, the guidelines for infective endocarditis, and the guidelines generally used in Japan (Additional File 1, Table S2). The duration of AG use was determined by referring to various medical practice guidelines.

The proportion of first-dose designs by pharmacists, the number of patients using AGs, the number of incidents of renal impairment, and the cost of AG use were also evaluated over time. Renal impairment was defined as a serum creatinine concentration increase of > 0.3 mg/dL or > 1.5-fold before and after AG administration [19] (Additional file 1, Table S3). The cost of AGs used was based on the drug prices as of 2023. Therefore, based on the formulation standards adopted by the hospital, the price per ampule was calculated as follows: tobramycin (60 mg), 403 yen; gentamicin (60 mg), 307 yen; amikacin (100 mg), 350 yen; and arbekacin (200 mg), 5750 yen. Any fractions were calculated as if one ampule had been used.

As a preliminary study, we interviewed five pharmacists routinely involved in AG dosing design and asked how much time was needed to determine AG dosing per patient.

### Statistical analyses

Binary variables were evaluated using Fisher’s exact probability test, and continuous variables were evaluated using the Mann–Whitney *U* test, both with a significance level of < 5%. EZR version 1.68 (Saitama Medical, Jichi Medical University, Saitama, Japan) was used for all analyses [20]. For missing data, statistical tests were performed by substituting the mean value of the item.

As a preliminary investigation, a factors analysis was performed in patients whose AG blood concentrations were determined to be completely manageable without dose adjustments. Logistic regression analysis assessed the factors that allowed for complete control of AG blood concentrations without dose adjustments. Factors included BMI, eGFR at the start of AG dosing, initial AG dose, and type of AG. Receiver operating curves were then drawn for significant factors and cut-off values based on Youden index were calculated.

## Results

### Patient background

This study included 123 patients before and 140 after the intervention and found no significant differences in patient backgrounds (Table 1). However, renal function was slightly worse in the post-intervention group than in the pre-intervention group (*P* = 0.080). There were no cases of low-dose AG use in combination with other antibacterial agents for synergistic effects against gram-negative bacteria or against urinary tract infections caused by gram-negative bacteria.

**Table 1:** Patient backgrounds

		Before intervention *N* = 123	After intervention *N* = 140	*P*-value
Aminoglycosides	Amikacin	47	59	0.26
	Gentamicin	46	54	
	Tobramycin	21	24	
	Arbekacin	9	3	
Characteristics	Male	80	93	0.90
	Age, years	69 (13)	68 (15)	0.67
Pre-aminoglycosides administration test values	Weight (kg)	53.6 (11.6)	53.8 (14.0)	0.69
	Body mass index (kg/m^2^)	21.1 (4.3)	20.9 (4.5)	0.87
	Creatinine (mg/dL)	0.75 (0.53)	0.92 (0.85)	0.07
	eGFR (mL/min/1.73 m^2^)	78.5 (23.3)	73.1 (24.7)	0.08
Focus of infection	Endocarditis	34	36	0.10
	Pneumonia	14	33	
	Skin and soft tissue	29	22	
	Febrile neutropenia	16	18	
	Others	28	26	
	Unknown	2	5	
Pathogens*	Gram-positive cocci	32	48	
	Streptococcus spp.	12	27	
	Staphylococcus spp.	8	14	
	Enterococcus spp.	9	6	
	Others	3	1	
	Gram-negative rods	30	43	
	* Pseudomonas aeruginosa* (Carbapenem resistant)	16	28	
	* Pseudomonas aeruginosa* (Carbapenem susceptible)	8	5	
	Enterobacterales (ESBLs)	2	4	
	Enterobacterales (non-ESBLs)	4	6	
	NTM	6	15	
	Others	2	1	
	Not detected	51	35	
	Multiple bacteria targeted	3	3	
	Aminoglycosides treatment duration	13 (10)	13 (9)	0.66

Presented as number of patients or means (standard deviations)

* Multiple bacterial infections were counted in duplicate

eGFR, estimated glomerular filtration rate; ESBL, extended-spectrum β-lactamase; NTM, non-tuberculous mycobacteria

### Impact on appropriate use

Table 2 shows the outcomes related to the appropriate AG use. Patients whose renal function was confirmed prior to AG treatment significantly increased from 93% pre-intervention to 99% post-intervention (odds ratio [OR], 9.67; 95% confidence interval [CI], 1.19–78.5; *P* = 0.01). Additionally, patients with confirmed weight significantly increased from 81% pre-intervention to 99% post-intervention (OR, 31.97; 95% CI, 4.25–240.66; *P* < 0.01). Twelve (10%) patients had a measured weight ≥ 20% of their IBW before the intervention and 14 (10%) after; all these patients were initially administered with appropriate doses without cases of renal impairment. The appropriateness of AG dosing considered based on infection foci and causative organisms was not significantly different, with 22% of patients considered non-recommended pre-intervention and 13% post-intervention (OR, 0.52; 95% CI, 0.27–1.01; *P* = 0.07). The proportion of patients for whom the initial AG dose was appropriate increased significantly from 74% pre-intervention to 91% post-intervention (OR, 3.44; 95% CI, 1.71–6.91; *P* < 0.01). Of the patients who used inappropriate AG doses, all were underdosed except for one patient pre-intervention. A total of 93% of patients pre-intervention and 95% post-intervention were judged to have used AGs for an appropriate duration, which was not significantly different (OR, 1.50; 95% CI, 0.54–4.16; *P* = 0.45). The proportion of patients meeting all of the above adequacy criteria increased significantly from 49% pre-intervention to 82% post-intervention (OR, 4.83; 95% CI, 2.76–8.44; *P* < 0.01).

**Table 2:** Changes in the rate of appropriate aminoglycoside use before and after intervention

	Before intervention *N* = 123	After intervention *N* = 140	*P*-value
**Required items for aminoglycoside administration**			
Renal function is assessed	115 (93%)	139 (99%)	0.01
Body weight is measured	100 (81%)	139 (99%)	< 0.01
**Appropriateness for the infection site and causative pathogen**			
Recommended	43 (35%)	59 (42%)	0.26
Optional	53 (43%)	63 (45%)	0.80
Not recommended	27 (22%)	18 (13%)	0.07
**Other appropriate use criteria**			
Initial dosage is appropriate	91 (74%)	127 (91%)	< 0.01
Duration of use is appropriate	114 (93%)	133 (95%)	0.45
Meets all criteria for appropriateness	60 (49%)	115 (82%)	< 0.01

### Impact on blood concentration management

During the study period, four measurement errors occurred, with either only the peak or trough concentration measured. Blood samples with these errors were excluded from the evaluation. The proportion of patients for whom the pharmacist performed the first dose design significantly increased from 21% pre-intervention (acceptance rate, 92%) to 60% post-intervention (acceptance rate, 97%) (OR, 2.49; 95% CI, 1.44–4.31; *P* < 0.01) (Table 3). The proportion of patients with measured AG blood concentrations significantly increased from 53% pre-intervention to 72% post-intervention (OR, 2.31; 95% CI, 1.39–3.85; *P* < 0.01); after 2020, AG blood concentrations were measured in 92% of patients. The median number of days to the first AG blood concentration measurement was significantly reduced from 4 (interquartile range, 3–6) days to 3 (2–4) days (*P* < 0.01). The proportion of patients who were able to manage target AG blood concentrations from the initial dose without dose adjustments increased from 31% pre-intervention to 42% post-intervention, although the results were not significantly different (OR, 1.63; 95% CI, 0.98–2.71; *P* = 0.07). Blood concentration analysis showed that 30% of the patients required dose adjustment pre-intervention and 37% post-intervention, with a nonsignificant difference. In addition, although not significantly different, more patients required AG dose increases before the intervention, and more patients required AG dose reductions after the intervention. There were 32 pre-intervention and 13 post-intervention patients with an initially underdosed AG dose. Of these, 21 pre-intervention and 8 post-intervention patients did not have AG blood concentrations measured. In addition, AG blood concentrations were measured in six pre-intervention and four post-intervention patients after the dose was corrected.

**Table 3:** Intervention outcomes of aminoglycoside blood concentration management and clinical endpoints

		Before intervention *N* = 123	After intervention *N* = 140	*P*-value
**Aminoglycoside blood concentration management**				
Initial dose recommendation by pharmacist		26 (21%)	84 (60%)	< 0.01
Patients who underwent blood concentration measurement		65 (53%)	101 (72%)	< 0.01
Number of days until the first blood concentration measurement		4 (3–6)	3 (2–4)	< 0.01
Number of blood concentration measurements		1 (0–1)	2 (0–2)	< 0.01
Patients who were able to maintain target blood concentration without dose adjustments		38 (31%)	59 (42%)	0.07
**Pharmacist intervention based on blood concentration analysis**				
Patients who required dose adjustment		37 (30%)^a^	46 (37%)	0.69
Dose increase^b^		15 (41%)	12 (26%)	
Dose reduction^b^		23 (62%)	34 (74%)	
**Dosage and Concentration**				
Amikacin^c^	First dosage (mg/kg/day)	7.1 (5.2–8.6)	8.2 (6.7–10.5)	0.04
	Dosage at end (mg/kg/day)	7.6 (6.1–9.8)	8.5 (7.4–12.8)	0.02
	Peak concentration (µg/mL)	34.7 (27.0–45.8)	46.9 (36.9–55.8)	< 0.01
	Trough concentration (µg/mL)	1.1 (0.9–2.0)	1.3 (0.8–2.1)	0.662
Gentamicin/ Tobramycin^d^	First dosage (mg/kg/day)	3.2 (2.0–3.9)	3.4 (2.4–4.8)	0.13
	Dosage at end (mg/kg/day)	3.0 (1.8–3.7)	3.9 (2.1–4.9)	0.14
	Peak concentration (µg/mL)	9.9 (8.1–15.9)	12.4 (9.3–16.5)	0.15
	Trough concentration (µg/mL)	1.1 (0.4–1.9)	0.6 (0.4–1.7)	0.19
Gentamicin for infective endocarditis^e^	First dosage (mg/kg/day)	2.3 (2.0–2.7)	2.6 (2.1–3.0)	0.37
	Dosage at end (mg/kg/day)	2.1 (1.3–2.9)	2.0 (1.4–2.5)	0.76
	Peak concentration (µg/mL)	4.5 (3.5–5.4)	6.3 (4.3–9.5)	< 0.01
	Trough concentration (µg/mL)	0.9 (0.6–1.2)	0.8 (0.4–1.0)	0.04
Arbekacin^f^	First dosage (mg/kg/day)	3.5 (2.4–5.0)	3.7 (3.6–5.3)	0.38
	Dosage at end (mg/kg/day)	3.5 (2.4–6.4)	5.5 (3.7–10.5)	0.27
	Peak concentration (µg/mL)	14.0 (11.6–17.2)	14.1 (9.3–20.2)	0.95
	Trough concentration (µg/mL)	0.6 (0.5–0.7)	0.9 (0.6–1.2)	0.38

Continuous variables are expressed as medians (interquartile ranges). For frequencies, the P-value was calculated using Fisher’s exact test, and for continuous variables, the Mann–Whitney U test was used

^a^ One patient required both a dose increase and reduction

^b^ Expressed as the proportion of patients who required dose adjustment

^c^ Blood concentration measurements were taken in a total of 32 pre-intervention and 70 post-intervention cases

^d^ Blood concentration measurements were taken in a total of 21 pre-intervention and 60 post-intervention cases

^e^ Blood concentration measurements were taken in a total of 50 pre-intervention and 80 post-intervention cases

^f^ Blood concentration measurements were taken in a total of 10 pre-intervention and 4 post-intervention cases

For all AGs, the median dose in the post-intervention was greater than pre-intervention. The median initial dose of AMK significantly increased from 7.1 (interquartile range: 5.2–8.6) mg/kg/day pre-intervention to 8.2 (6.7–10.5) mg/kg/day post-intervention (*P* = 0.04); doses were also significantly different at the end of treatment (before vs. after, 7.6 [6.1–9.8] vs. 8.5 [7.4–12.8] mg/kg/day; *P* = 0.02). Similarly, the median peak AG blood concentration post-intervention was greater than pre-intervention for all AGs. Significant differences in peak blood concentrations were observed for AMK and GM for infective endocarditis. In addition, there was a significant decrease in trough concentrations for GM for infective endocarditis, although no significant changes were found for the other AG uses.

Factor analysis was performed on 166 patients whose AG blood concentrations were completely controlled without the need of AG dose adjustments. No significant differences were found in body mass index (BMI), initial AG dose, and type of AG. However, eGFR at the start of AG dosing was extracted as a significant factor (OR: 1.03, 95% CI: 1.01–1.04, *P* < 0.01). The area under the receiver operating characteristic curve was 0.69 (95% CI: 0.60–0.78), with a sensitivity of 91% and specificity of 44%; the sensitivity increased with increasing eGFR. Using Youden’s index, the optimal eGFR cut-off was 54.3 mL/min/1.73 m2, suggesting that patients with an eGFR better than this cut-off value may achieve better controlled AG blood concentrations without AG dose adjustments.

### Intervention outcomes on clinical endpoints

The clinical outcomes are shown in Table 4. In total, 65% of patients had “effective” AGs before the intervention and 71% after (OR, 1.30; 95% CI, 0.77–2.18; *P* = 0.35). Discontinuation of AGs due to adverse effects was 4.9% before and 7.1% after the intervention (OR, 1.5; 95% CI, 0.53–4.25; *P* = 0.61). A total of 11% of the patients before and 12% after the intervention had decreased renal function (OR, 0.91; 95% CI, 0.42–1.97; *P* = 0.85). The mortality rates were 4.9% pre-intervention and 3.6% post-intervention (OR, 0.72; 95% CI, 0.21–2.43; *P* = 0.76). None of these differences were statistically significant.

**Table 4:** Intervention outcomes for clinical endpoints

	Before intervention *N* = 123	After intervention *N* = 140	*P*-value
**Clinical efficacy**			
Effective	80 (65%)	99 (71%)	*P* = 0.35^a^
Not effective	30 (24%)	29 (21%)	
Unnecessary use	13 (11%)	12 (8.6%)	
**Adverse events**			
Renal impairment^b^	13 (11%)	17 (12%)	*P* = 0.85
Discontinuation due to adverse events	6 (4.9%)	10 (7.1%)	*P* = 0.61
Kidney injury	5 (4.1%)	3 (2.1%)	*P* = 0.48
Ototoxicity	0 (0%)	1 (0.7%)	*P* = 1.00
Skin rash	0 (0%)	2 (1.4%)	*P* = 0.50
Hepatic injury	0 (0%)	2 (1.4%)	*P* = 0.50
Diarrhea	1 (0.8%)	0 (0%)	*P* = 0.47
Loss of appetite	0 (0%)	1 (0.7)	*P* = 1.00
Death	6 (4.9%)	5 (3.6%)	*P* = 0.76

^a^ Comparison between effective and other items

^b^ Cases, wherein renal function was measured before and after the use of aminoglycosides (115 patients before intervention and 127 patients after intervention) were targeted

### Change in AG use over time

**Fig. 1:**
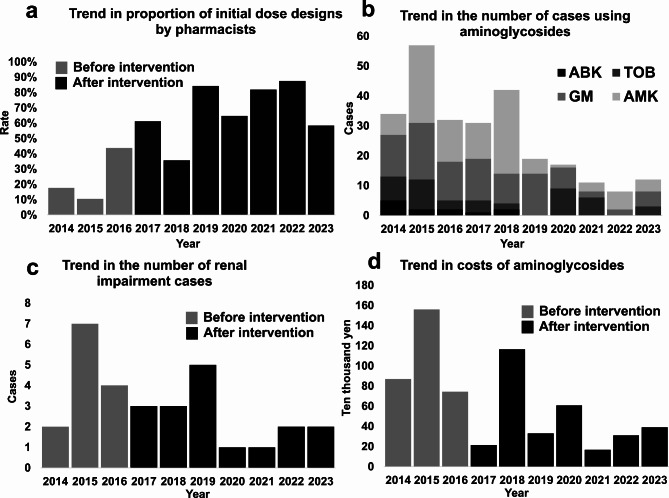
Annual trends in the impact of a pharmacist-led intervention on aminoglycoside use. (**a**) The rate of initial dose designs by pharmacists increased over time. (**b**) There was a decrease in the number of patients using AGs and in the number of AGs used over time. (**c**) The number of patients with renal impairment showed a decreasing trend with the continuation of the intervention. (**d**) The amount of aminoglycoside drugs used gradually declined. ABK, arbekacin; AG, aminoglycoside; AMK, amikacin; GM, gentamicin; TOB, tobramycin

Changes in AG use over time are depicted in Fig. 1. The annual average number of patients using AGs decreased from 41 pre-intervention to 21 post-intervention, although the difference was not statistically significant (*P* = 0.07). The mean number of days of AG administration decreased from 535 days/year pre-intervention to 279 days/year post-intervention (*P* = 0.12), which was not a significant decrease. The annual mean number of renal impairment cases decreased from 4.3 pre-intervention to 2.5 post-intervention, which was nonsignificant (*P* = 0.24). The average amount spent on AGs decreased by 56% from 1,060,000 yen/year pre-intervention to 470,000 yen/year post-intervention (*P* = 0.07). During the interview, five pharmacists claimed that the time required to design an AG dose per patient was about 5–15 min.

## Discussion

This study demonstrated that pharmacist-led AG stewardship contributes to their appropriate use. Additionally, the results of this long-term observational study indicate that continued intervention increases consultations with pharmacists at the time of initial AG administration. This increases the opportunity to discuss the need for AGs, suggesting that this may lead to a reduction in AG use.

TDM is an important routine practice for maximizing the efficacy of antimicrobials and minimizing their adverse effects. Pharmacists are important professionals involved in supporting the proper use of antimicrobials and should perform TDM as a routine practice [12]. Although TDM is essential for AG use, only 4–20% of facilities routinely perform TDM due to a lack of time and workforce to support the proper use of antimicrobial agents [13, 14, 21]. A possible reason for this is the low frequency of use. Patients administered less frequently used medications are less likely to be educated about their proper use, making it difficult for them to take hold. In the present study, AG stewardship by pharmacists significantly contributed to their proper use at the time of initial administration. One of the reasons AGs may be underdosed is the gap between labeling and guideline recommendations. For example, the Japanese package inserts for AMK states “100–200 mg intravenously twice daily” (available at https://www.nichiiko.co.jp/medicine/file/55270/attached_pdf/55270_attached.pdf　2024.09.06). However, the recommended AMK dose reported in recent years is “15–20 mg/kg every 24 h” [22, 23]. Houot et al. reported that interventions involving only semi-passive dissemination of recommendations were insufficient to promote appropriate AG use [24]. Support for the appropriate use of AGs by pharmacists has demonstrated that AGs can play a role in bridging the far-flung dose gap. As inadequate doses of antimicrobials can lead to reduced clinical efficacy, pharmacist-led interventions may improve clinical outcomes. In addition, patients with better renal function appeared to have better control of AG blood concentrations in the preliminary factor analysis of this study, suggesting that for those with impaired renal function, the use of alternative medications with stronger recommendations may decrease the likelihood of renal impairment.

By examining a long-term intervention over a 7-year period, we were able to observe its impact over time. Although there have been previous reports of reduction in AG medications, most have been based primarily on aggressive interventions by physicians trained in infectious diseases [25]. Therefore, little is known about the potential of TDM-centered interventions to increase consultations with pharmacists. In the current intervention, pharmacist involvement was not mandatory for the first dose of AGs, as it could be administered at the physician’s discretion. However, as the duration of the intervention increased, consultations with the pharmacist at the time of initial administration increased. Our hospital has a system in which a pharmacist specializing in infection control is available for consultation. One of the barriers to TDM implementation is the lack of expert or specialized training [26]. A situation where trained pharmacists are monitoring AG drugs, as in the present study, may indicate that these barriers can be removed. Additionally, Cook et al. reported a 91.3% reduction in AG prescriptions after 13 years of antimicrobial stewardship, highlighting the importance of continued intervention, complementing the results of the present study, which demonstrated the importance of continuing the intervention [27]. The time spent supporting the proper use of infrequently used AGs is not that long (5–15 min per case). Interventions, such as the one assessed herein, are a reasonable method for facilities with few resources and can generate sufficient value, even when only the cost of reducing usage is considered. Additionally, controlling the use of AG drugs is expected to reduce the drug resistance rate; therefore, a secondary cost-reduction effect can also be expected [28]. This study may have overestimated drug cost reductions due to potential shifts from AGs to more expensive alternatives. However, since the use of AGs deemed “not recommended” declined post-intervention, cost reductions were likely partially realized. Moreever, several factors may have contributed to reduced antimicrobial use. During the study, there were no resistant gram-negative rod outbreaks and the susceptibility rate of *Pseudomonas aeruginosa* to AMK remained above 90%. Besides pharmacist involvement, the decrease in AG use may also reflect reassessment of AGs for infective endocarditis and open fractures [17, 29].

The most important limitation of the present study was the heterogeneity of patient backgrounds. For example, GM combination therapy for infective endocarditis is not comparable to AMK combination therapy for nontuberculous mycobacterial infections. Further, Roger et al. reported no improvement in the clinical outcomes with the use of AGs for septic shock, even at appropriate AG blood concentrations [30]. Due to the infrequent use of AGs and the small sample size, it was impossible to conduct a study with uniform patient backgrounds. Moreover, optimizing AG administration leads to the maximization of efficacy and minimization of side effects. However, in the present study, there was no significant improvement in clinical efficacy or safety pre- and post-intervention. In the future, it will be necessary to verify the effect of the intervention on a uniform number of target patients. In the present study, no clear difference was observed in the incidence of renal impairment between the pre- and post-intervention periods. Renal impairment caused by AGs mainly correlates with trough blood concentration but may also be related to the area under the curve [31, 32]. Avoiding underdosing by implementing the current intervention led to an increase in the area under the curve; however, this intervention did not lead to a decrease in renal function. Moreover, the number of patients with reduced renal function tended to decrease as the number of patients receiving AGs decreased with appropriate AG blood concentration control. There are few high-quality reports on the target blood concentrations of AGs [33]. Therefore, clinical feedback on the target values provided in the guidelines and other sources is required.

## Conclusions

This study was a multifaceted analysis of the impact of a pharmacist-led TDM intervention based on criteria developed using the information accumulated to date. The results revealed a reduction in AG use and optimization of AG blood concentration management. The findings also suggest that this intervention may improve safety. Consequently, pharmacists should provide support for the appropriate use of AGs whenever possible.

## Electronic supplementary material

Below is the link to the electronic supplementary material.

Supplementary Material 1: Definition of the criteria for assessing the appropriateness of aminoglycosides. Table S1. Appropriate initial dose and serum concentration of aminoglycosides based on renal function; Table S2. Appropriateness of aminoglycoside use; Table S3. Clinical endpoints.

## Data Availability

The datasets generated and analyzed during the current study are available from the corresponding author upon reasonable request.

## Abbreviations

AG: Aminoglycoside
BMI: body mass index
TDM: Therapeutic drug monitoring
AMK: Amikacin
GM: Gentamicin
IBW: Ideal body weight
OR: Odds ratio
CI: Confidence interval
