# Supplementary material for: Impact of pharmacist-led aminoglycoside stewardship: a 10-year observational study
Source: J Pharm Health Care Sci. 2024 Nov 28;10:77. doi: 10.1186/s40780-024-00399-x (PMC11605850; doi:10.1186/s40780-024-00399-x)
Supplement: Supplementary file 1 — Supplementary Material 1: Definition of the criteria for assessing the appropriateness of aminoglycosides. Table S1. Appropriate initial dose and serum concentration of aminoglycosides based on renal function; Table S2. Appropriateness of aminoglycoside use; Table S3. Clinical endpoints. [file 40780_2024_399_MOESM1_ESM.docx]

**Impact of Pharmacist-Led Aminoglycoside Stewardship: A 10-Year Observational Study**

*Journal of Pharmaceutical Health Care and Sciences*

Yasutaka Shinoda, Kengo Ohashi, Tomoko Matsuoka, Kaori Arai, Nao Hotta, Eiseki Usami

Department of Pharmacy, Ogaki Municipal Hospital, Gifu, Japan

Corresponding Author: Yasutaka Shinoda

Email Address: [shonoda065039@gmail.com](mailto:shonoda065039@gmail.com)

**Additional file 1: Definition of the criteria for assessing the appropriateness of aminoglycosides**

**Table S1.** Appropriate initial dose and serum concentration of aminoglycosides based on renal function

|  | GM/TOB | | AMK | | ABK |
| --- | --- | --- | --- | --- | --- |
| eGFR  mL/min/1.73 m^2^ | MIC=2 μg/mL | MIC≤1 μg/mL | MIC=8 μg/mL | MIC≤4 μg/mL |  |
| ≥80 | 7 mg/kg, every 24 h | 5 mg/kg, every 24 h | 20 mg/kg, every 24 h | 15 mg/kg, every 24 h | 5 mg/kg, every 24 h |
| 70–79 | 5 mg/kg, every 24 h | 4 mg/kg, every 24 h | 15 mg/kg, every 24 h | 12 mg/kg, every 24 h | 4 mg/kg, every 24 h |
| 60–69 |  |  |  | 7.5 mg/kg, every 24 h |  |
| 50–59 | 4 mg/kg, every 24 h | 3.5 mg/kg, every 24 h | 12 mg/kg, every 24 h |  | 3.5 mg/kg, every 24 h |
| 40–49 |  | 2.5 mg/kg, every 24 h |  | 4 mg/kg, every 24 h | 2.5 mg/kg, every 24 h |
| 30–39 | 5 mg/kg, every 48 h |  | 15 mg/kg, every 48 h |  |  |
| 20–29 | 4 mg/kg, every 48 h | 4 mg/kg, every 48 h | 12 mg/kg, every 48 h | 7.5 mg/kg, every 48 h | 4 mg/kg, every 48 h |
| 10–19 | 3 mg/kg, every 48 h | 3 mg/kg, every 48 h | 10 mg/kg, every 48 h | 4 mg/kg, every 48 h | 3 mg/kg, every 48 h |
| Target serum concentration | | | | |  |
| Peak | ≥15–20 μg/mL | ≥8–10 μg/mL | 50–60 μg/mL | 41–49 μg/mL | ≥15 μg/mL |
| Trough | ＜1 μg/mL | | ＜4 μg/mL | | <1–2 μg/mL |

ABK, arbekacin; AMK, amikacin; MIC, minimum inhibitory concentration; eGFR, estimated glomerular filtration rate; GM, gentamicin; TOB, tobramycin

Gentamicin administration for infective endocarditis was deemed appropriate at an initial dose of 3 mg/kg/day. In this case, a peak concentration of ≥3 μg/mL and a trough concentration of <1 μg/mL were considered appropriate.

When used at low doses in combination with other antimicrobial agents for synergistic effects against gram-negative bacteria, gentamicin or tobramycin at 3 mg/kg for 24 h and amikacin at 400 mg for 24 h were considered appropriate. For urinary tract infections caused by gram-negative bacteria, gentamicin or tobramycin at 3 mg/kg for 24 h and amikacin at 10 mg/kg for 24 h were also considered appropriate. In both cases, peak concentrations were not clearly defined, but trough concentrations were evaluated.

In this study, adjusted body weight was used to assess dose adequacy in patients with ≥20% of their ideal body weight (IBW), as follows: IBW = A + 0.91 x [height – 152.4], where A is 50 for men and 49.5 for women; and adjusted body weight (kg) = IBW + [0.4 x (actual body weight - IBW)].

Based on adopted formulation standards and clinical practices, appropriate dose tolerances were less than ±100 mg for amikacin and less than ±60 mg for gentamicin, tobramycin, and arbekacin.

References: Sanford Guide to Antimicrobial Therapy and TDM Guidelines developed by the Japanese Society of Chemotherapy

**Table S2.** Appropriateness of aminoglycoside use

|  | Clinical definition | Example | Duration | References |
| --- | --- | --- | --- | --- |
| Recommended | 1. Listed as a first-line drug in global guidelines 2. No alternatives available | ⚫ Combination therapy with gentamicin for prosthetic valve endocarditis of Gram-positive cocci | 2 weeks  2 to 6 weeks for Enterococci | [1, 2] |
|  |  | ⚫ Combination therapy for *Mycobacterium abscessus* infections | 4 to 12 weeks | [15] |
|  |  | ⚫ Infections caused by Gram-negative bacteria sensitive only to aminoglycosides | Within 2 weeks^a^ | [16] |
| Optional | 1. Listed as a secondary option in guidelines, or not explicitly mentioned but used when other drugs are ineffective 2. Use of aminoglycosides is justified based on the literature | ⚫ Combination with cefazolin for open fractures (Gustilo classification III or higher) | Within 24 h of wound closure  If the wound cannot be closed within 72 h | [17] |
|  |  | ⚫ Febrile neutropenia not responding to carbapenem therapy | Until clinically recovered and afebrile for 72 h | [18] |
|  |  | ⚫ Combination therapy for *Pseudomonas aeruginosa* resistant to standard treatment | Within 2 weeks^a^ | [16] |
| Not recommended | 1. Safer and more effective alternatives, including β-lactam antibiotics, are available 2. Use without identifying the infection focus | ⚫ Infections caused by Enterobacteriaceae with good susceptibility | Within 2 weeks^a^ | [16] |
|  |  | ⚫ Administration based solely on the presence of fever |  |  |

^a^Follow guidelines for deep abscesses

In addition to these, the Sanford Guide to Antimicrobial Therapy was used as a reference (<https://www.sanfordguide.com/>, 2024.9.8).

**Table S3.** Clinical endpoints

|  | Terms | Definition |
| --- | --- | --- |
| Clinical effectiveness | Effective | ⚫ Fever has resolved, localized infection has improved, or cultures are negative, and antimicrobial therapy has been completed  ⚫ Improvement in inflammatory markers (procalcitonin, C-reactive protein, white blood cell count) and completion of the guideline dosing period (e.g., 2 weeks of gentamicin for streptococcal endocarditis) |
|  | Not effective | ⚫ Persistent fever or local findings of infection (e.g., sputum or decreased oxygenation in the case of pneumonia) and a change from an aminoglycoside to another antibacterial or death  ⚫ Failure to complete the dosing period specified in the guidelines due to adverse events |
|  | Unnecessary use | ⚫ When the source of the organism or the focus of infection is identified, and the patient is switched from an aminoglycoside to another antimicrobial  ⚫ Intent to use and intent to terminate are unknown |
| Adverse events | Renal impairment | Serum creatinine level increased by >0.3 mg/dL from the start to the end of aminoglycoside use, or if the level increased by ≥1.5. Reference no. [19] |
